# The 10th Oxbridge varsity medical ethics debate-should we fear the rise of direct-to-consumer genetic testing?

**DOI:** 10.1186/s13010-018-0069-9

**Published:** 2018-10-29

**Authors:** Christian Michael Armstrong Holland, Edward Harry Arbe-Barnes, Euan Joseph McGivern, Ruairidh Mungo Connor Forgan

**Affiliations:** 1 Trinity College, University of Oxford, Oxford, UK; 2 Magdalen College, University of Oxford, Oxford, UK; 3 Oriel College, University of Oxford, Oxford, UK; 4 Corpus Christi College, University of Cambridge, Cambridge, UK

**Keywords:** Genetic testing, Direct-to-consumer testing, Genetics, Genomics

## Abstract

In an increasingly data-driven age of medicine, do companies that offer genetic testing directly to patients represent an important part of personalising care, or a dangerous threat to privacy? Should we celebrate this new mechanism of patient involvement, or fear its implications?

The Universities of Oxford and Cambridge addressed these issues in the 10th annual Medical Ethics Varsity Debate, through the motion: “This House Regrets the Rise of Direct-to-Consumer Genetic Testing”. This article summarises and extends key arguments made in the debate, exploring the impacts of such genetic testing on both the individual patient and broader society, with special consideration as to whether companies can ever truly guarantee anonymity of genetic data.

## Background

Direct-to-consumer (DTC) genetic testing refers to any form of genetic testing sold directly to consumers without the involvement of a medical professional. Consumers are sent a buccal swab as part of a kit, which they return through the post. This sample is then analysed for several thousand different single nucleotide polymorphisms (SNPs) at various genomic loci, in order to provide information about the individual’s genetic constitution [1].

The market for such tests is competitive, and currently occupied by a variety of different companies, such as 23andMe, Atlas Biomed and EasyDNA [2–4]. It is currently estimated as worth $140 million worldwide and projected to be worth around $340 million by 2022 [5]. While tests were originally marketed mainly to provide information about an individual’s ancestry and family history, over recent years they have become increasingly medically-focussed. Many companies now highlight the ability to identify an individual’s genetic susceptibility to a range of diseases, one such example being 23andMe’s screening for BRCA 1 and 2, noting their predictive value in breast and ovarian cancer [6–8].

In determining the burden of proof for this debate, we considered the impact of these tests on the individual consumer and on society as a whole, with regards to both medical benefits and potential threats to privacy. In order to claim whether we should regret the rise of such testing or not, opposing sides were required to demonstrate either overall net harm or benefit in relation to both the individual and society.

Thus, the debate and this re-analysis of the relevant issues, centre on these three questions:

1. Is DTC testing beneficial or harmful to individual users?
2. Is DTC testing beneficial or harmful to society as a whole?
3. Do DTC tests represent a threat to privacy?

## Question 1-is DTC testing beneficial or harmful to individual users?

In asking whether DTC tests represent a net good or harm to the individuals who choose to use them, it is reasonable to start by considering what the manufacturing companies claim.

Atlas Biomed and EasyDNA, for example, suggest that their clients will derive some degree of satisfaction through learning about their own genomes, but also market strongly on the potential for disease prevention through diet and lifestyle changes [4, 9].

The first claim is reasonable. People who choose to undergo genetic testing at their own volition and expense might well be expected to gain some degree of intellectual satisfaction or entertainment value from doing so, and in learning something about their own genetic makeup. Indeed, available evidence supports this claim. 59% of a Canadian cohort considering DTC testing claimed to be motivated by curiosity [10], while 80% of a post-test cohort described themselves as having gained satisfaction from the test [11].

This certainly provides a benefit in favour of DTC tests: they provide direct satisfaction and entertainment to consumers through assuaging curiosity and providing a point of interest. This point was accepted without challenge by both sides in the debate.

However, evidence also shows that 82% of people considering DTC genetic tests wanted to know their risk status for heritable diseases [12]. Similarly 93% claim they’d use the test to change their lifestyle if they found themselves at a higher risk of disease and 80% wanted to inform their children of their genetic risks and to allow their doctor to monitor their health more closely [13].

Importantly, the Canadian cohort provides the only head-to-head comparison of patients’ reasons for purchasing DTC tests. While 59% of the group were motivated by curiosity when considering genetic tests, 95% were interested in their risk for developing medical conditions. Furthermore all were more interested in purchasing genetic tests relating to more serious genetic conditions as opposed to less serious and were willing to pay more for these [10].

Therefore we must also consider the claim that such testing will encourage individuals to improve their diet and lifestyles.

As an example, Atlas Biomed tests for predisposition to 17 multi-factorial diseases, including type II diabetes mellitus (T2DM), hypertension and obesity [14].

While the company doesn’t reveal exactly which exact genes have made it onto their panel, it is clear from the literature that they have carefully selected diseases with complex and common heritability. For example 30–50% of the variability in blood pressure is due to genetic heritability and up to 50% of individuals with hypertension have genetic factors that contribute to this [15]. Many of these genes are involved in the renin-angiotensin-aldosterone system, and are found scattered across chromosomes 7, 8, 18 and 21 [16, 17].

Similarly while there are some monogenic causes of obesity, generally involving the hormone leptin and its receptor, these are rare: being present in fewer than 5% of patients with severe obesity [18]. Far more common are a range of SNPs with smaller effect sizes clustered in this pathway, that contribute to the genetic component of non-monogenic obesity [19, 20]. T2DM presents a similar picture: with over 70 susceptibility genes contributing to its heritability.

Supporters of DTC testing argue that individuals thus informed of a greater risk of developing these diseases might increase their weekly exercise and improve their diet in response: the personal information provided by DTC testing might provide a direct spur to addressing modifiable risk factors.

Of course the converse argument is also possible. It is entirely conceivable that other patients, given a much better genetic prognosis, might take it as justification for adopting a rather more laissez faire attitude to diet and exercise.

Upon turning to the evidence to ask which of these responses to testing is more likely we find a thoroughly equivocal response. When a cohort of 2240 patients were followed up a year after their genetic testing there was no change in their fat intake or exercise from baseline levels pre-testing [11], suggesting that neither the proposition nor opposition could claim this point in the debate.

A much more important argument however is the question of whether DTC testing could reveal an individual’s genetic susceptibility for potentially life-threatening conditions such as cancer, through screening for BRCA 1 and 2, and what the implications of such knowledge would be. The debate came to hinge on this question.

Such screening carries two obvious benefits, which we will illustrate with the example of BRCA screening.

Firstly, knowledge of an increased susceptibility to breast and ovarian cancer will allow a patient to act pre-emptively. For example, prophylactic mastectomy and salpingo-oophorectomy significantly reduce the risk of developing breast and ovarian cancer respectively [21, 22]. Thus DTC testing informs patients of relevant risks, and allows them to act to reduce these.

Secondly, even if patients choose not to undergo such risk-reducing surgery, they will be more aware of their chances of developing such cancers. Thus they can inform themselves of the relevant signs and symptoms and choose to undergo regular screening in order to detect any such cancer as soon as possible. Given that the efficacy of cancer treatment is enhanced by early detection [23, 24] DTC testing might reasonably be expected to increase life expectancy.

However, we would argue that this is a simplistic view of testing, and needs more thorough consideration. Although the BRCA genes provide important information about an individual’s risk of cancer, they need to be interpreted in the context of an individual’s family history and other risk factors: including alcohol intake, age at menarche and age at menopause [25]. Such interpretation is an important medical role, normally fulfilled by a clinical geneticist, and the fact that DTC testing provides genetic information without concurrent medical interpretation and advice, provides several definite harms.

The first such harm is that information, without concurrent interpretation and medical advice, is likely to generate distress and anxiety in those tested. It is one thing to be told that you have a 70% chance [6, 7] of developing cancer, by a doctor who has been trained to break bad news well and who then provides immediate advice on potential risk-reducing interventions or available screening programmes. It is another to receive the same news in a paper report from an anonymous company, run by medically unqualified staff and which does not provide either potential treatment or advice concerning the results.

Thus while supporters of DTC testing suggest that it allows patients to address their particular health risks directly, this hinges on access to appropriate medical advice and treatment. Without this, we argue that such screening can only be harmful: generating anxiety by informing patients of risks of serious diseases about which they can do very little.

A range of evidence supports this position. In 2014 Boeldt et al. showed that people who received DTC test results showing a higher risk for diseases such as colon cancer or Alzheimer’s disease had increased anxiety after testing [26]. Similarly others who received DTC test results showing an increased risk of developing alcoholism showed a decrease in mood and increase in anxiety afterwards [27].

In the case of breast cancer, a recent case report proves enlightening. Dohany and colleagues describe a woman who learned she had a BRCA mutation through a genetic test and subsequently developed severe anxiety, distress and insomnia [28]. Importantly, she only recovered after several sessions of genetic counselling, during which she planned a risk reducing salpingo-oophorectomy along with breast surveillance. She reports that her ability to address her increased risk through surgery and intensive surveillance, along with the genetic counselling, was key in her recovery.

This is supported by work from Francke and colleagues in 2013, who followed a group of 16 women after they received similar BRCA diagnoses [29]. While 4 initially developed similar levels of anxiety, all recovered well upon accessing genetic counselling and planning surgery to reduce their risk of cancer.

Therefore, while we can argue that DTC screening will initially provide a large degree of anxiety upon receipt of results, it is also important to assess how individuals will access further care, as this can be vital in preventing post-test anxiety. We must also consider whether this is harmful at an individual and societal level.

In states with privately funded healthcare, such as the USA, a particular set of problems emerge when we consider individuals with a level of income sufficient to purchase DTC testing but insufficient to provide further medical care. While tests now cost around $100–150 [3, 4], access to medical services can be far more expensive than this, and risk-reducing surgery is even more costly, with risk-reducing mastectomies costing between $16,000 and $44,000 [30].

Therefore, in this case, DTC clearly fails to fulfil Wilson’s criteria for an effective screening programme [31]: if the patient cannot access risk-reducing surgery for their greater risk of breast cancer then it is undesirable to screen for such a risk.

At the individual level this harm is reflected in the continued anxiety the patient is likely to experience: knowing they have a high risk of developing cancer without any option to reduce that risk. This clearly qualifies as a net harm to the individual as a result of DTC testing.

## Question 2-is DTC testing beneficial or harmful to society as a whole?

To answer this second question we can apply the above hypothetical BRCA patient to a state funded healthcare system, such as the UK’s National Health Service (NHS).

Here the patient has immediate and free access to medical services through their GP. Presenting this doctor with evidence of a greatly increased risk of cancer will be sufficient to gain referral to secondary care without any additional cost.

The patient will therefore be able to access both the necessary genetic interpretation and, if their risk is sufficiently great, risk-reducing mastectomy or other relevant treatments.

Whether this constitutes a net good or harm to society however, hinges on a deeper ethical question.

The allocation of resources in the NHS is determined by organisations such as The National Institute for Health and Clinical Excellence (NICE). These exist to maximise the improvement in quality and quantity of life that can be generated with public funding: approving interventions that generate the maximum gain in years of quality life (QALYs) per unit funding [32]. Screening for BRCA is already available in the NHS for patients with a pre-test probability of having a BRCA 1 or 2 mutation of 10% or more, as estimated from family history [33].

It was argued during the debate that when a patient accesses a GP service and is referred for further assessment and treatment because of results obtained privately, they are consuming resources which otherwise would have been spent on patients purely on the basis of clinical need and maximising utility. It might then be argued that DTC testing provides a route for patients to access NHS services on the basis of their ability to pay an initial £150 cost. This therefore subverts the role of organisations such as NICE, instead causing a redistribution of NHS resources to those with a greater ability to pay.

The contrasting view is that by taking the financial burden of screening upon themselves, patients actually increase the number of QALYs purchased per unit expenditure by the NHS, as the health service is presented with BRCA positive patients, and can begin counselling and treatment immediately. By contrast, the cost of screening 10 patients for each one who is found to be BRCA positive (given a pre-test probability of 10%) increases the cost per QALY bought if the NHS is also responsible for the screening.

Is DTC testing beneficial or harmful to society as a whole? The answer hinges on a second question - is an increase in QALYs per unit of NHS expenditure a benefit if it comes through redistribution of QALYs to the disproportionately wealthy? Do we value overall QALYs obtained, or equality of access to those QALYs?

The redistribution of NHS resources on the basis of ability to pay for DTC testing is fundamentally opposed to the founding principle of the service: that care should be provided on the basis of clinical need alone rather than the ability to pay. The subversion of this principle by allowing access to services through privately obtained results could therefore be seen as unethical: a mechanism by which DTC tests and their sequelae are harmful to society.

However, the principle of equality of access at the core of the NHS is in turn based on ideas of promoting equality across society as a whole. In broad terms this is desirable because the psychological and health harms of inequality can be so severe. Greater societal inequality is associated with increased obesity [34], shorter life expectancy, reduced health [35] and increased mortality [36].

In order to analyse whether the form of inequality that DTC testing generates will lead to these harms, we first need to understand exactly how they derive from inequality. Why is it that an unequal society gives rise to unhappy and unhealthy people?

Classically sociological arguments contend that economic inequality leads to increased mistrust and status competition. Such effects then induce negative effects on health and well-being [37].

Inequality leads to a generalised distrust of others, with perception that the economic system is unfair. Such a breakdown in trust between individuals contributes to shorter life expectancy because the breakdown of social groups leads to less healthy lives [38]. In particular degradation in trust and cooperation between individuals strongly correlates with levels of depression and other mental health conditions [39–42].

These psychological effects of inequality derive from the processes outlined in social comparison theory. Individuals compare social standing on criteria such as financial resources, where there are few objective standards, in order to build a perception of their own status [43]. Via the status-anxiety hypothesis, individuals make upwards comparisons between themselves and others: those who perceive themselves as inferior experience discomfort [44]. This is consistent with data that individuals are unsatisfied when neighbours are wealthier [45] or if average pay in their occupation is higher [46]. Satisfaction with one’s position is heavily dependent on relative comparisons [47]. Vitally it is thus relative comparisons that lead to the negative psychological effects of inequality.

Therefore, when we consider the inequality generated by users of DTC tests accessing NHS services we can use the arguments outlined above to determine whether it is likely that significant harms will accrue.

In summary, the harms of inequality are mainly derived from a perception of inequality, resulting in breakdown of social trust and generation of mental health problems. Therefore, to generate these harms the inequality must be readily visible: people must be widely aware of DTC testing, and must perceive the advantage that it gives to those able to afford it.

Yet, knowledge of these tests and what they do is not universal and we argue that they are not flagrant enough breaches of NHS principles to result in any significant public outcry that would lead to greater awareness.

There are two broad reasons to support this position. Firstly public awareness of these tests in the first place is very low: only around 13–14% of people are aware of their existence [13, 48]. Secondly, it is a large jump from knowing of the existence of these tests to fully considering the implications of their use in terms of QALYs and NHS funding. There is certainly no evidence of this argument being made at the national level. A Google search of ‘DTC testing inequality’ yields no UK news articles raising concerns about the inequality harms of DTC testing.

Even in the literature surrounding DTC testing we believe we are the first to make this argument. PubMed searches of ‘DTC testing inequality’ and ‘Direct to consumer testing inequality’ returned 16 results in total, none of which made any reference to this argument.

It is reasonable that this could change in the future if large amounts of inequality were made public by concerted press involvement. This would, however, rely on the difficult conversion of an unaware public.

Under the status quo, access to NHS services on the back of DTC testing is simply not a form of inequality of which many people are aware and about which people care significantly. Thus we should not expect it to generate significant levels of the negative effects of inequality outlined above.

Therefore, while this testing does undermine the aim of the NHS not to discriminate on the basis of the ability to pay, it is not incompatible with the principles behind that aim: and will not generate significant harms of inequality.

Thus, by generating an increase in QALYs gained per unit of NHS expenditure, without generating significant associated harms DTC tests represent a net benefit to society.

It is also important to note that every case of potential breast cancer that is treated prophylactically on the basis of a DTC test is one fewer that is detected at a more advanced stage. Not only does this result in greater life expectancy, it also saves resources: as treatment at a later stage involves not only surgery but also chemotherapy and/or radiotherapy, with far more extensive medical follow up. Therefore more resources are saved at this later stage through earlier diagnosis and treatment.

## Question 3-do DTC tests represent a threat to privacy?

A final question that bridges impacts on both society and the individual is whether DTC genetic testing represents a dangerous threat to privacy.

While DTC companies frequently promise that all genetic data remains anonymous, recent research questions whether that is ever possible.

Based on the principle that human Y chromosomes almost always segregate with surname, as in almost all societies children take their father’s surname, several databanks now exist that are able to pair Y chromosome sequence data with likely surname. Using this principle, along with other publically available data such as birth records, Gymrek and colleagues were able to identify 50 supposedly anonymous individuals whose genomes were available in public datasets in a proof of principle paper in 2013 [49]. Projection of their methods predicts that the same approach would be able to identify around 13% of individuals from their genome data.

This raises important questions about the privacy of genetic data held by DTC testing companies, and whether it could be used to disadvantage those who have undergone the testing. Clearly knowledge of an individual’s susceptibility to life-threatening diseases would be of great interest to health, life and disability insurance companies. In the USA the former are legally prevented from discriminating on the basis of genetic data, by the Genetic Information Nondiscrimination Act of 2008 (GINA). Life and disability insurance companies suffer no such restrictions however [50].

Clearly this concern is speculative: we are not aware of any reports of genetic data being used in such a way to date.

In analysing whether this is a likely risk in the future there are two main considerations.

Firstly, it is possible that the damaging publicity associated with exploiting such legal loopholes might well discourage DTC companies from doing so. If it became known that they were profiting from the sale of genetic data that was used to directly disadvantage their consumers, then they could be at risk of seriously reduced sales. Indeed the fact that they sell directly to these consumers increases the importance of a positive public image.

Secondly, even if such exploitation of genetic data were to become commonplace, it would be possible for states to amend existing legislation to prevent this. Mechanisms could include a requirement to leave key regions of the Y chromosome un-sequenced, or an expansion of the uses of data currently outlawed.

However, as noted above, this analysis is speculative: given that this potential harm of DTC testing has not yet materialised it is difficult to predict the likely outcomes. Indeed the second point above raises the interesting question of how quickly any state legislature could respond to emergent technologies. An early version of GINA was considered by congress in 1995 [51], but it took 13 years before the legislation was actually passed [52].

It might be possible that such legislation could be amended in less time than it took to pass originally, but there is the potential for serious harm to accrue through the misuse of genetic data while states scramble to keep up with private companies.

In summary, while Gymrek’s work shows it is often impossible to anonymise an individual’s complete genome this advance has so far remained unexploited, and future legislation could conceivably be prepared to protect privacy. However it is impossible to predict the likelihood of these prospective events with any certainty, and thus to judge truly how serious the threat to individual privacy from DTC testing is.

## Conclusions

DTC tests undoubtedly provide a source of interest and enjoyment to those who choose to use them, and represent a speculative threat to privacy. However their most important impacts revolve around screening for disease.

In this context we argue that whether they represent a net good or harm hinges on the funding of healthcare in the jurisdiction in which they are used. Where DTC tests can act as an adjunct to an existing state funded healthcare system such as the NHS, they represent an excellent mechanism for generating an increase in years of quality life, through early diagnosis and treatment. Importantly, harms relating to inequality at the societal level are likely to be minimal. By contrast, where healthcare is privately funded DTC tests represent a much less effective screening tool: likely generating significant anxiety and harm, without providing a route to effective and accessible treatment.

Therefore the impacts of DTC tests cannot be analysed outside the societal context in which they arise, and hence our titular question has two answers. If healthcare is privately funded DTC tests are likely to represent a net harm; in state-funded systems the converse is true.

### About the debate

The inaugural Varsity Medical Ethics Debate in 2008 continued the centuries-old rivalry between the Universities of Oxford and Cambridge, and has run since then as an annual event hosted alternately at the respective Unions. This year The Cambridge Union played host to the 10th annual debate, with an audience of medical students, doctors and academics in attendance.

After consideration of the arguments raised by both sides the judges awarded the victory to Oxford who, interestingly, argued the opposite case to that made in this article. Both sides however were able to enjoy a broad and thorough consideration of the relevant issues, and will follow the development of such testing over the next few years with great interest.

## Abbreviations

DTC: Direct to consumer
GINA: Genetic Information Nondiscrimination Act
NHS: National Health Service
NICE: National Institute for Health and Clinical Excellence
QALY: Quality adjusted life year
SNP: Single nucleotide polymorphisms
